# P-394. Antiretroviral therapy persistence following a change or restart in regimen among people with HIV

**DOI:** 10.1093/ofid/ofaf695.611

**Published:** 2026-01-11

**Authors:** Benjamin Chastek, Uche Mordi, Lisa B Le, Seojin Park, Cassidy Trom, Travis Lim, Mary J Christoph

**Affiliations:** Optum, Eden Prairie, Minnesota; Gilead Sciences, Inc., Foster City, CA; Optum, Eden Prairie, Minnesota; Gilead Sciences, Inc., Foster City, CA; Gilead Sciences, Inc., Foster City, CA; Gilead Sciences, Inc., Foster City, CA; Gilead Sciences, Inc., Foster City, CA

## Abstract

**Background:**

Describe and compare regimen persistence for people with HIV (PWH) after switching or restarting antiretroviral therapy (ART) overall and among Medicare Advantage (MA) enrollees.

**Figure:**
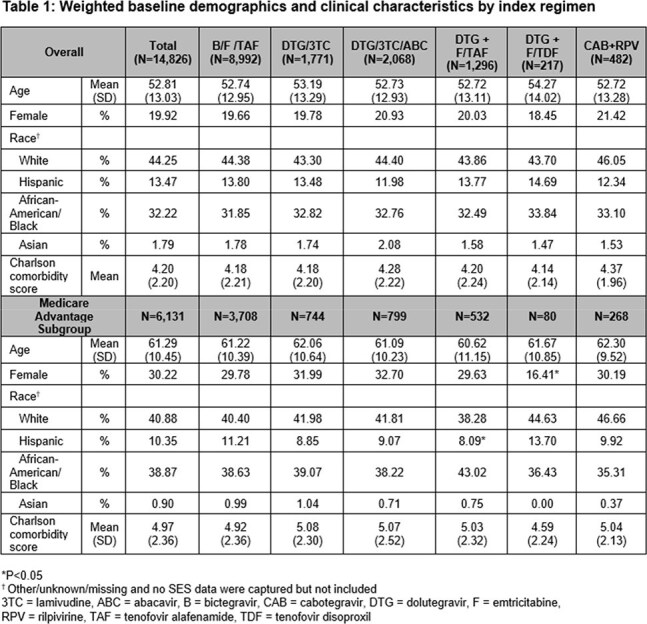

**Figure:**
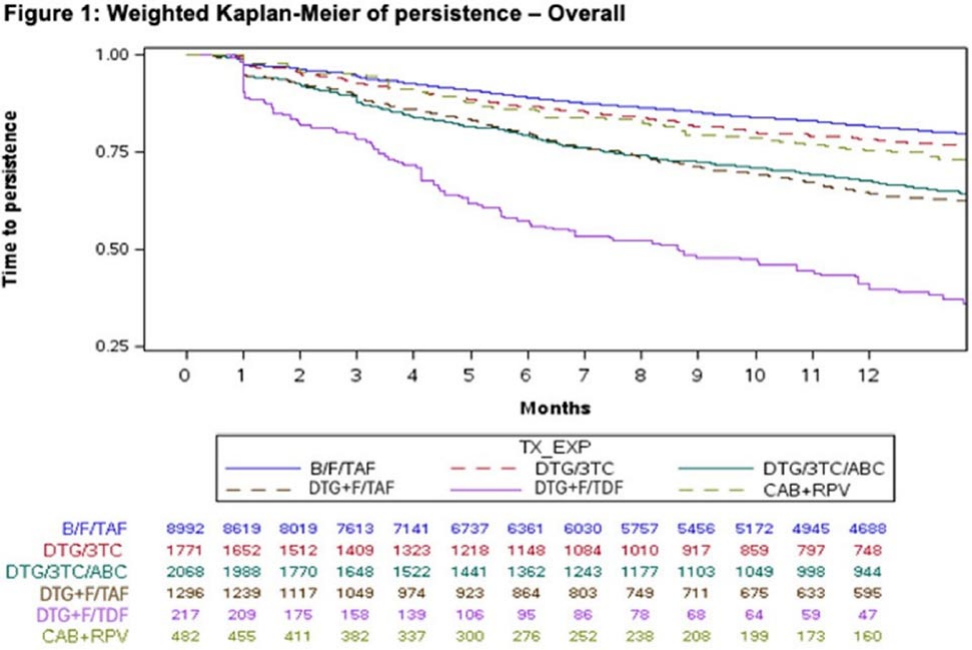

**Methods:**

This retrospective study used medical and pharmacy claims data for patients with commercial health insurance or MA with Part D coverage (Optum Research Database). The index line of therapy was identified for patients switching or restarting ART between 07/01/2017 – 11/30/2023. Persistence was defined as the time to the earliest of ART discontinuation (gap in all ART ≥90 days), ART switch or add on, death, or end of available data. Outcomes were evaluated for: DTG/ABC/3TC, B/F/TAF, DTG/3TC, DTG + F/TDF, DTG + F/TAF and CAB+RPV. Analysis was conducted overall and among MA enrollees. Inverse Probability Treatment Weighting was implemented to adjust for demographic characteristics, baseline clinical measures, and baseline health care cost and utilization. Kaplan-Meier analysis was conducted after weighting examining the effect of regimen selection on ART persistence at 1 year.

**Figure:**
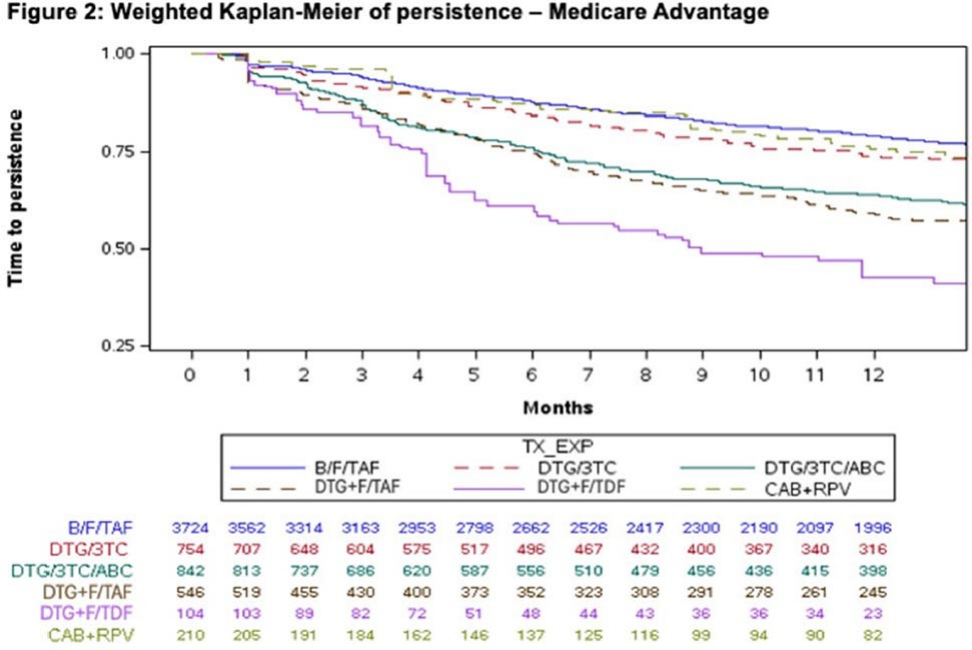

**Results:**

A total of 113,705 individuals had a diagnosis of HIV, and of those, 14,344 either switched or restarted ART. After weighting, mean age was 53 years, 20% of PWH were female, 44% were white, and 42% were enrolled in a MA plan (Table 1).

From weighted Kaplan-Meier analysis of both the overall sample and the MA subgroup, the percent of PWH persistent at 1 year was significantly greater (P< 0.05) for B/F/TAF vs. DTG/3TC, DTG/3TC/ABC, DTG+F/TAF and DTG+F/TDF and numerically greater but not statistically different vs CAB+RPV though comparisons cannot be reliably calculated with CAB+RPV due to the limited available sample size (Figure 1 and 2).

**Conclusion:**

Treatment experienced PWH starting B/F/TAF were more likely to be persistent at 1 year compared to other commonly used ART regimens. Similar results were observed among the Medicare Advantage subgroup despite older age and greater burden of comorbidities.

Funding: Gilead Sciences Inc.

**Disclosures:**

Benjamin Chastek, MS, Optum (UnitedHealth Group): Stocks/Bonds (Public Company) Uche Mordi, PharmD, MS, Gilead Sciences, Inc.: Stocks/Bonds (Public Company) Seojin Park, PharmD, MS, Gilead Sciences, Inc.: Employee at the time of the development Cassidy Trom, PharmD, AAHIVE, Gilead Sciences, Inc.: Employee|Gilead Sciences, Inc.: Ownership Interest|Gilead Sciences, Inc.: Stocks/Bonds (Public Company) Travis Lim, MSc, DrPH, Gilead Sciences, Inc.: Employee|Gilead Sciences, Inc.: Stocks/Bonds (Public Company) Mary J. Christoph, PhD, MPH, AstraZeneca: Advisor/Consultant|AstraZeneca: Employee|AstraZeneca: Stocks/Bonds (Public Company)|Gilead Sciences, Inc.: Employee|Gilead Sciences, Inc.: Stocks/Bonds (Public Company)

